# The Antibacterial Synthetic Flavonoid BrCl-Flav Exhibits Important Anti-*Candida* Activity by Damaging Cell Membrane Integrity

**DOI:** 10.3390/ph14111130

**Published:** 2021-11-06

**Authors:** Cornelia Babii, Mihaela Savu, Iuliana Motrescu, Lucian Mihail Birsa, Laura Gabriela Sarbu, Marius Stefan

**Affiliations:** 1 Biology Department, Faculty of Biology, The Alexandru Ioan Cuza University of Iasi, Bld. Carol I, Nr. 11, 700506 Iasi, Romania; corneliababii@yahoo.ro (C.B.); mihaelasavu2@gmail.com (M.S.); 2 Sciences Department, Research Institute for Agriculture and Environment, Iasi University of Life Sciences, 3 Sadoveanu Alley, 700490 Iasi, Romania; imotrescu@yahoo.com; 3 Faculty of Chemistry, The Alexandru Ioan Cuza University of Iasi, Bld. Carol I, Nr. 11, 700506 Iasi, Romania; lbirsa@uaic.ro

**Keywords:** anti-*Candida*, synthetic flavonoid, antifungal agents, mechanism of action, synergistic effect

## Abstract

Infections caused by *Candida* are very difficult to treat due to increasing antifungal resistance. Recent studies showed that patients with *Candida* infections resistant to fluconazole have very few treatment options. Therefore, finding new efficient antifungal agents is a matter of medical high priority. The aim of this study was to explore the antifungal potential of BrCl-flav-a representative of a new class of synthetic flavonoids with bromine as halogen substituent at the benzopyran core against four *Candida* clinical strains. Determination of minimum inhibitory concentration and minimum fungicidal concentration along with the time kill assay indicated a strong antifungal effect of BrCl-flav against *C. albicans*, *C. parapsilosis*, *C. krusei* and *C. glabrata*. The investigation of anti-*Candida* mechanism of action using fluorescence microscopy and scanning electron microscopy revealed that Br-Cl flav could inhibit fungal growth by impairing the membrane integrity, the resulting structural damages leading to cell lysis. BrCl-flav also showed important anti-virulence properties against *Candida* spp., inhibiting biofilm formation and yeast to hyphal transition. A strong synergistic antifungal effect against *C. albicans* strain was observed when BrCl-flav was used in combination with fluconazole. BrCl-flav has a good potential to develop new effective antifungal agents in the context of *Candida* spp. multidrug resistance phenomenon.

## 1. Introduction

*Candida* pathogenic species are considered a major cause of morbidity and mortality worldwide, representing a serious threat to public health [1]. The most frequently isolated pathogenic species is *C. albicans*, causing infections which can cause 40–60% mortality, especially in immunocompromised patients [2]. Other species such as *C. glabrata*, *C. tropicalis*, *C. parapsilosis* and *C. krusei* have been also isolated and are considered the main causative agents in 50–70% of systemic fungal infections [1,3].

Antifungal agents such as azoles, polyenes, echinocandins, nucleoside analogs and allylamines are used to control *Candida* infections. Among the available anticandidal agents, fluconazole (a type of azole) is one of the most frequently used drugs for the treatment of *Candida* infections [4]. However, antifungal chemotherapy is challenged by multidrug resistance, phenomenon extensively documented in the literature among *Candida* species, especially in relation to azoles [1]. One of the major factors behind the multiple drugs resistance is the ability of *Candida* spp. to form biofilms on different surfaces [5]. Due to their resistance to multiple drugs, biofilm cells survive to antifungals concentrations 1000-fold higher than those which inhibit planktonic cells [6]. Therapeutical limitations caused by multidrug resistance phenomenon make the control of *Candida* infections a major challenge for modern medicine. Therefore, the design of new drugs derived from the traditional ones and the identification of new antifungal molecules are fundamental to face the alarming increase of *Candida* infections rate [1].

Flavonoids, a group of natural heterocyclic compounds, are potentially good candidates for developing new antifungal agents. Traditionally, flavonoids have been used for centuries to treat human diseases due to their antimicrobial, anti-allergic, anti-inflammatory and antioxidant activities. The antifungal properties of natural flavonoids are well documented [7]. Nowadays, antifungal research is focused on semisynthetic and synthetic flavonoids due to their higher antimicrobial activity [7,8,9,10]. However, at this moment little is known about antifungal potential of synthetic flavonoids [8].

In this study, a representative of a new class of synthetic sulfur containing tricyclic flavonoids with bromine as halogen substituent at the benzopyran core (BrCl-flav) was tested *in vitro* for antifungal activity against four *Candida* clinical strains: *C. albicans*, *C. parapsilosis*, *C. krusei* and *C. glabrata*. We previously showed that BrCl-flav is an effective antibacterial agent, with stronger bacteriostatic and bactericidal effect at lower concentrations than those described in the earlier reports [11]. Based on its potent antibacterial properties, BrCl-flav has a good potential in developing new effective antifungal agents in the context of *Candida* spp. multidrug resistance phenomenon. Nothing is known so far about the BrCl-flav antifungal activity, therefore we focused on BrCl-flav mechanism of action and the effect in combination with fluconazole against *C. albicans* resistant clinical isolates. The novelty of the present work is related to the proposal of a synthetic flavonoid—BrCl-flav as a new antifungal agent with good potential to develop effective drugs in the context of *Candida* spp. multidrug resistance phenomenon.

## 2. Results

### 2.1. Antifungal Activity of Synthetic Flavonoid

BrCl-flav showed a promising inhibitory activity against all tested *Candida* isolates at concentrations as low as 15.62 μg/mL and fungicidal activity at a concentration of 31.25 μg/mL (Table 1).

#### 2.1.1. BrCl-Flav Inhibited *Candida* spp. Growth in a Dose and Time Dependent Manner

**Figure 1:**
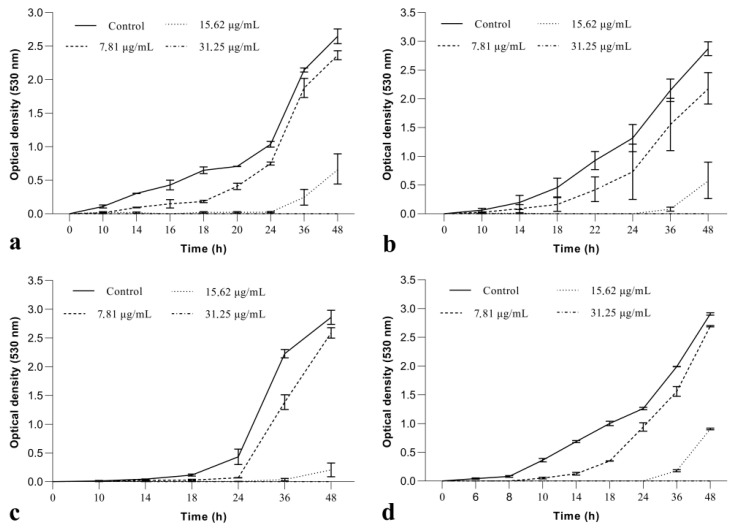
Growth kinetics of *Candida* spp. treated with BrCl-flav at different concentrations: (**a**) *C. albicans*; (**b**) *C. krusei*; (**c**) *C. parapsilosis*; (**d**) *C. glabrata*. Bars indicate standard deviations (*p* < 0.05).

The MIC value (15.62 µg/mL) was used as reference to evaluate the effect of BrCl-flav on fungal growth. As the growth dynamics curves depicted in Figure 1 show, no significant inhibition was recorded when BrCl-flav was used at a concentration of 7.81 μg/mL (½ MIC). When used at a concentration equivalent to MIC, BrCl-flav significantly inhibited the growth of all clinical isolates (*p* < 0.05) for more than 24 h compared with control. The growth of *Candida* fluconazole-resistant strains was suppressed up to 48 h at 2 × MIC, showing that BrCl-flav has an important fungistatic activity. The growth curves also revealed a progressive inhibition of the fungal growth by increasing the concentrations of the tested agent in the SDB medium.

#### 2.1.2. BrCl-Flav Has a Potent Fungicidal Activity against *Candida* spp.

**Figure 2:**
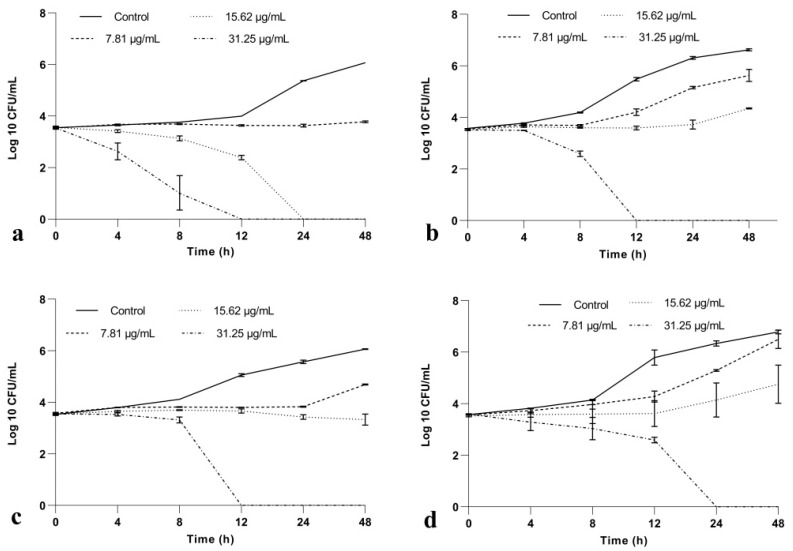
Time-kill curves after BrCl-flav exposure at different concentrations: (**a**) *C. albicans*; (**b**) *C. krusei*; (**c**) *C. parapsilosis*; (**d**) *C. glabrata*. Bars indicate standard deviations (*p* < 0.05).

Time-kill assay showed a reduction in the number of viable *Candida* spp. cells for all BrCl-flav tested concentrations (Figure 2). Thus, a significant reduction in microbial growth (*p* < 0.013) of <3 log10 in CFU/mL compared with the initial inoculum (considered as a fungistatic effect) was recorded when fungal cells were exposed more than 12 h to BrCl-flav at concentrations equivalent to MIC. A fungicidal effect (a reduction in microbial growth of ≥3 log10 in CFU/mL) was evidenced after exposing *Candida* spp. cells to BrCl-flav concentrations equivalent to 2 × MIC. Also, we must emphasize that BrCl-flav at 2 × MIC induced total kill (no viable cells) after only 12 h of incubation against fluconazole resistant strains (*C. albicans*, *C. krusei*) and *C. parapsilosis* strain, respectively.

### 2.2. BrCl-Flav Mode of Action

#### 2.2.1. Complexation of Sorbitol

MIC values of BrCl-flav did not change or were slightly lower (no statistically significant differences) in the presence of sorbitol, compared to BrCl-flav tested alone, as the results presented in Table 2 show.

#### 2.2.2. Exposure to BrCl-Flav Caused Cellular Membrane Damage

**Figure 3:**
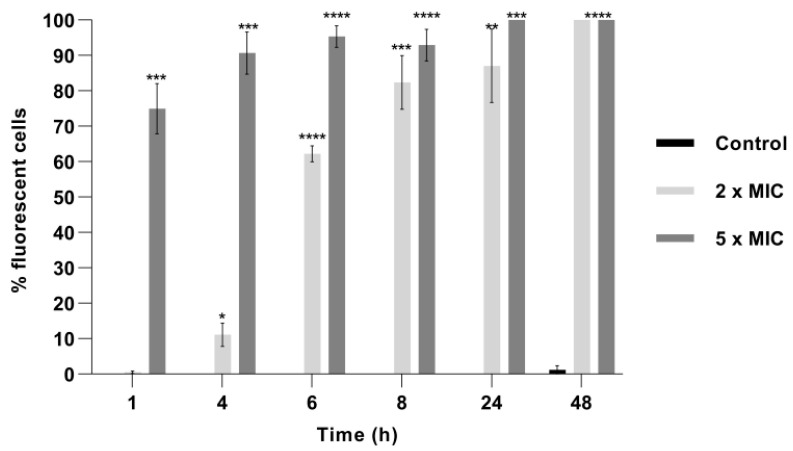
Effect of BrCl-flav on *Candida albicans* cell membrane structure. Cells were treated with concentrations of BrCl-flav equivalent to 2 × MIC and 5 × MIC and stained with propidium iodide. Bars indicate standard deviations. Asterisk represents a significant difference (*p* < 0.05) vs. Control (* = *p* < 0.0476; ** = *p* < 0.0019; *** = *p* < 0.0008; **** = *p* < 0.0001).

**Figure 4:**
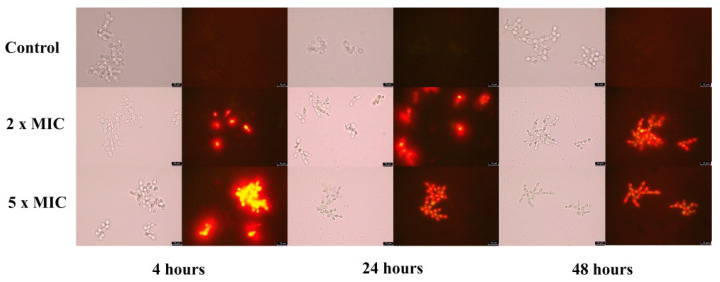
Effect of the BrCl-flav exposure on membrane permeability of *Candida albicans* exponential-phase cells to propidium iodide. Red fluorescence cells were detected in samples using fluorescence microscopy after 4, 24 and 48 h of incubation with BrCl-flav (concentrations equivalent to 2 × MIC and 5 × MIC); magnification 1000×.

Penetration of PI into dead or injured *C. albicans* cells exposed to BrCl-flav was evidenced using fluorescence microscopy. The number of fluorescent cells significantly increased in time in a dose dependent manner after BrCl-flav treatment (*p* < 0.047) at concentrations equivalent to 2 × MIC, starting with 4 h of incubation (Figure 3). After 48 h all BrCl-flav exposed cells were fluorescent (Figure 4). Increasing the BrCl-flav concentration to 5 × MIC induced more significant membrane damage. Thus, after only 1 h of incubation, 75% of the *C. albicans* cells treated with BrCl-flav were fluorescent and after 24 h of exposure the percentage increased to 100%. No fluorescent cells were detected in control up to 48 h, showing the inability of PI to penetrate viable cells with intact plasma membranes (Figure 3).

#### 2.2.3. BrCl-Flav Induced Irreversible Morphological Damage

**Figure 5:**
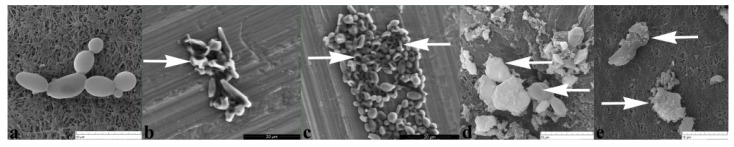
SEM photomicrographs showing the effects of BrCl-flav on *Candida albicans* cell morphology: (**a**) control; (**b**) cells exposed 6 h to MIC; (**c**) cells exposed 6 h to 2 × MIC; (**d**) cells exposed 6 h to 5 × MIC; (**e**) cellular debris. Arrows indicate irreversible morphological damage of treated fungal cells. These scanning electron photomicrographs are representative of a typical result.

**Figure 6:**
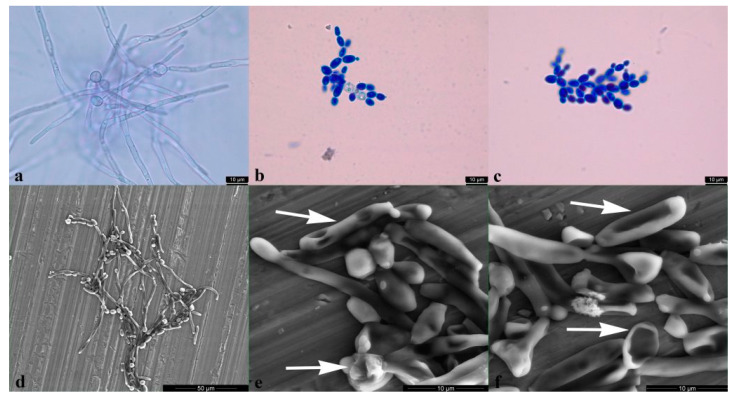
BrCl-flav inhibition of *C. albicans* hyphal formation in liquid RPMI 1640 (6 h at 37 °C): control cells (**a**,**d**); cell exposed to BrCl-flav at concentrations equivalent to MIC (**b**,**e**) and 2 × MIC (**c**,**f**); control cells formed hyphae (**a**,**d**); hyphae formation was inhibited by BrCl-flav at MIC (**e**); yeast to hyphal transition was prevented by BrCl-Flav at 2 × MIC equivalent concentration (**c**,**f**); treated cells appeared colored in blue when a methylene blue staining was performed to distinguish between live and dead cells (**b**,**c**) and showed significant morphological damages (**e**,**f**). Arrows indicate irreversible morphological damage of treated fungal cells. Images were obtained using a light microscope, magnification 1000× (**a**–**c**) and SEM (**d**–**f**).

Scanning electron microscopy was employed to assess the effect of BrCl-flav on *C. albicans* cell morphology. Control cells showed an intact morphology, with regular, smooth surface and a clear boundary (Figure 5a). The analysis of SEM photomicrographs pointed out that BrCl-flav exposure resulted in considerable morphological damage of fungal cells compared with control, along with release of inner cell materials, most probably due to cell lysis and aglutination, as it can be seen in Figure 5b–e and Figure 6e,f.

#### 2.2.4. *Candida albicans* Yeast to Hyphal Transition Was Prevented by BrCl-Flav

The effect of BrCl-flav at concentrations equivalent to MIC and 2 × MIC on *C. albicans* hyphal formation was evaluated using hypha-inducing conditions (incubation in RPMI 1640 medium). The results showed that BrCl-flav significantly inhibited yeast to hyphal transition starting with 6 h incubation time. We must emphasize that the treated cells did not formed hyphae in the presence of all tested BrCl-flav concentrations up to 48 h (data not shown), except for MIC samples for which the inhibition rate calculated at 6 h was 57.14%. When a methylene blue staining was performed to distinguish between live and dead cells, all 2 × MIC BrCl-flav exposed cells appeared colored in blue, being considered as dead (Figure 6c). The BrCl-flav inhibition of hyphae formation was also confirmed by SEM. The photomicrograph presented in Figure 6f show both the absence of hyphae and irreversible morphological damage of fungal cells incubated in the presence of BrCl-flav at concentrations equivalent to 2 × MIC.

#### 2.2.5. BrCl-Flav Impedes *Candida* spp. Biofilm Formation

**Figure 7:**
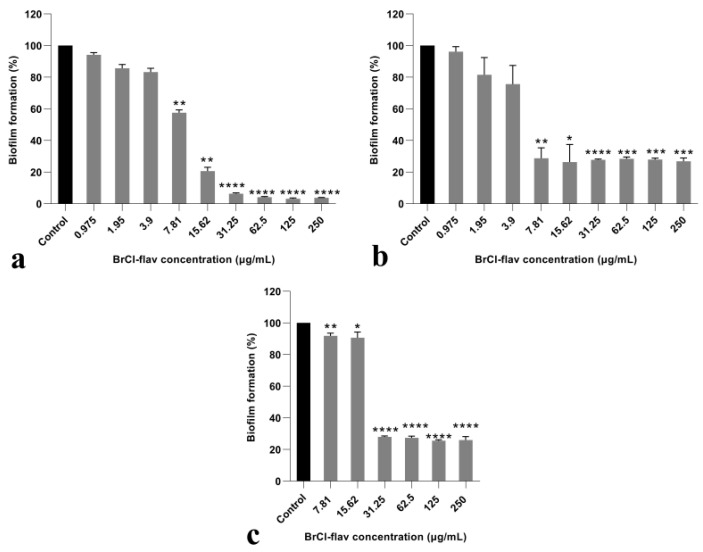
In vitro activities of BrCl-flav against *Candida* spp. biofilm formation: (**a**) *C. albicans*; (**b**) *C. krusei*; (**c**) *C. glabrata*. Bars indicate standard deviations. Asterisk represents a significant difference (*p* < 0.05) vs. Control (* = *p* < 0.0435; ** = *p* < 0.0088; *** = *p* < 0.0009; **** = *p* <0.0001).

Anti-biofilm activity was assessed against three *Candida* strains: *C. albicans*, *C. krusei* and *C. glabrata*. Our results showed that BrCl-flav had a significant inhibitory effect on biofilm formation for all tested strains, in a dose-dependent manner (Figure 7). A significant reduction in biofilm formation (*p* < 0.0030) up to 80% compared to control was evidenced at concentrations equivalent to MIC for *C. albicans* and *C. krusei*. Also, it should be noted that significant antibiofilm activity (*p* < 0.0088) was recorded for both strains at subinhibitory BrCl-flav concentrations (¼ MIC and ½ MIC).

### 2.3. Effect of BrCl-Flav in Combination with Fluconazole against Candida Strains

Assessment of the combination between BrCl-flav and fluconazole against *Candida* strains showed that the tested combinations produced synergistic, additive and indifferent interaction effects (Table 3). 

The only synergistic interactions (FICI ≤ 0.5) were recorded for *C. albicans* strain resistant to fluconazole. When used in combination, the MIC values of the two agents were reduced 128-fold for fluconazole and up to 8-fold for BrCl-flav.

**Figure 8:**
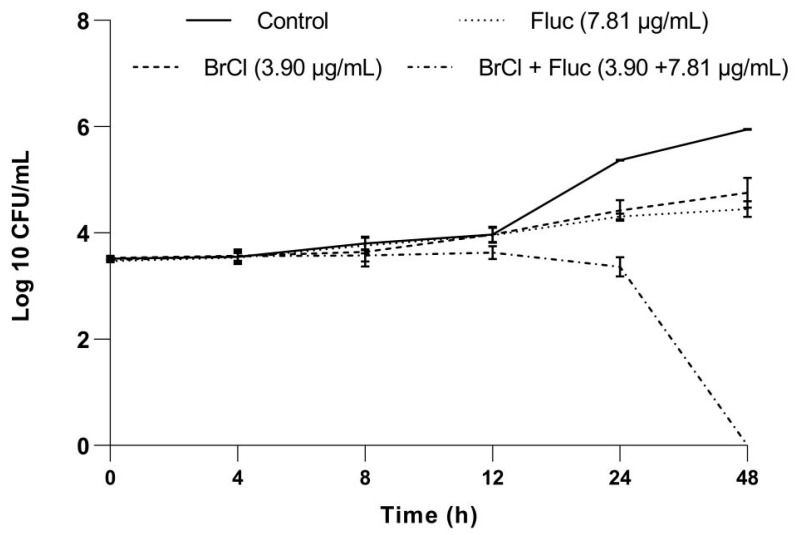
The time-kill curve of BrCl-flav and fluconazole synergistic combination against *Candida albicans* strain. Bars indicate standard deviations (*p* < 0.05).

A combination of BrCl-flav and fluconazole (3.9/7.81 µg/mL) was further selected to study the synergistic effect over time, using a time-kill assay. No reduction in the number of viable *Candida albicans* cells was recorded for BrCl-flav and fluconazole used alone, compared with the control. However, when used in combination, the two tested antifungal agents showed a significant fungicidal activity (*p* < 0.0012), with total kill after 48 h (Figure 8).

## 3. Discussion

Infections caused by fungi like *Candida* are very difficult to treat due to increasing antifungal resistance. Recent studies showed that patients with *Candida* infections resistant to fluconazole have in fact very few treatment options (US Centers for Disease Control and Prevention, https://www.cdc.gov/fungal/diseases/candidiasis/antifungal-resistant.html (accessed on 3 November 2021)). Moreover, patients who have drug-resistant *Candida* bloodstream infections are less likely to survive than patients who have infections caused by drug-sensitive *Candida* strains [12,13]. Therefore, finding new efficient, low toxicity antifungal agents is a matter of medical high priority.

BrCl-flav is a synthetic tricyclic flavonoid representative for a novel class of sulfur containing flavonoids easy and simple to obtain in a cost-effective manner. We previously showed that this compound has a remarkable antibacterial activity at low concentrations: 0.24 µg/mL against *Staphylococcus aureus* and 3.9 µg/mL against *Escherichia coli* [11]. The main mechanism responsible for the bactericidal effects is related to the impairment of the cell membrane integrity and cell lysis. As such, we have strong reasons to consider that BrCl-flav has a good potential for the development of new antimicrobial agents. Therefore, further studies using four clinical *Candida* isolates were employed to investigate BrCl-flav antifungal properties and mechanism of action.

A potent antifungal effect was evidenced for BrCl-flav against all *Candida* strains tested *in vitro* using the determination of minimum inhibitory concentration. The method allowed us to assess the lowest concentration of BrCl-flav which inhibited the growth of the tested fungal strains—15.62 μg/mL. When compared with the reference drug fluconazole, a considerable higher antifungal activity was recorded against fluconazole resistant *C. albicans*, *C. krusei* and *C. glabrata* isolates. Compared to most of the reported natural and synthetic flavonoids, our compound displayed a stronger antifungal activity, being up to 28-fold more active against *Candida* spp. (e.g., baicalein—MIC up to 21 μg/mL; myricetin—MIC up to 64 μg/mL; quercetin—MIC up to 441 mg/mL, etc) — [7,14,15]. We must emphasize that BrCl-flav exhibited an antifungal activity comparable to some chalcone, flavones and flavanones derivatives considered to be the most potent synthetic flavonoids against *Candida* reported so far (MIC values ranging from 1 to 16 μg/mL)—[7].

The same important antifungal activity of BrCl-flav was evidenced by the growth kinetics studies. The fungistatic effect was dose-dependent, increasing concentrations of BrCl-flav progressively inhibited the fungal growth of all tested *Candida* isolates. The cells incubated with 7.81 µg/mL BrCl-flav (concentration equivalent to ½ MIC) showed no significant growth inhibition compared with control. On the other hand, a significant growth delay (up to 28 h) represented by prolonged lag phases occurred when *Candida* spp. cells were incubated in the presence of BrCl-flav at 15.2 µg/mL, corresponding to MIC. We must point out that no turbidity was recorded by spectrophotometric measurements for all *Candida* cells exposed to 31.25 µg/mL within the time span of the experiments (48 h), denoting a strong fungicidal activity.

A time-kill kinetics assay was employed to determine the fungistatic or fungicidal activity of BrCl-flav over time. The analysis confirmed also the important antifungal activity of BrCl-flav against all *Candida* spp. after exposure to concentrations equivalent to MIC (fungistatic effect) and 2 × MIC (fungicidal effect). One exception occurred for *C. albicans* strain—a fungistatic effect was recorded by the growth experiments up to 24 h, while time kill studies revealed a fungicidal effect (99.9% killing of cells) at 24 h. A reasonable explanation could be related to different experimental conditions used for the two assays. We must emphasize that no viable cells (total kill) were detected starting with 12 h after BrCl-flav exposure at 31.25 µg/mL (equivalent to 2 × MIC), suggesting an important fungicidal potential. The fact that this activity has been recorded against fluconazole-resistant *Candida* strains makes BrCl-flav a more interesting compound for practical applications. Our conclusion is supported by a comparative literature survey which revealed that BrCl-flav has higher fungicidal activity (up to 48-times) compared to many natural flavonoids [16,17,18]. Also, the recorded activity was higher or comparable with other synthetic flavonoids such as different chalcone, 1,3-thiazole and 2-hydrazinyl-1,3-thiazole derivatives [8,19,20,21]. We must emphasize that BrCl-flav acted as a more potent fungicidal compared with fluconazole, considered to be a gold standard of antifungal agents [22].

Sorbitol binding affinity assay (MIC determination in the presence and absence of 0.8 M sorbitol) was used to investigate the interference of BrCl-flav with the *Candida* cell wall. Sorbitol is an osmotic protector which can support cell growth when the fungal wall is targeted by antifungal agents. In the absence of sorbitol, the fungal growth is inhibited due to the disruption of the cell wall. The effect is detected by an increase of the MIC value in the presence of sorbitol compared to the MIC value determined in medium without sorbitol. [23]. In our study, MIC values of BrCl-flav did not change in the presence of sorbitol, suggesting that the tested synthetic flavonoid does not target the cell wall. Therefore, other potential cellular targets were investigated. Penetration of PI into dead or injured *C. albicans* cells was evidenced using fluorescence microscopy. PI is a cell membrane-selective permeable dye that can only pass through damaged or permeabilized cell membranes, binding to DNA and exhibiting characteristic red fluorescence [24]. Our results revealed a gradual increase of the fluorescent cells number with the increasing concentration of the tested antifungal. After 24 h all cells exposed to BrCl-flav at a concentration equivalent to 5 × MIC were fluorescent, suggesting that the tested antifungal significantly damaged the fungal cell membrane integrity. Usually, damages to the cell membrane are related to cell lysis. SEM image analysis showed severe alteration of the cell morphology, with collapsed cells, wrinkled surfaces, along with cellular debris resulting from the disintegration of the BrCl-flav treated cells. These morphological changes are most likely caused by cell lysis. All those results clearly indicate that the main BrCl-flav mechanism of action is related to membrane disruption followed by cell lysis.

Hyphae and biofilm formation—two critical virulence factor of *C. albicans*—were investigated to better understand how BrCl-flav affects fungal cells. Both *Candida* virulence attributes are interlinked, contributing not only to the host tissue invasion but also to the evasion of host immunity [17]. Our study revealed that BrCl-flav prevented both yeast to hyphal transition and biofilm formation. The effect was concentration dependent, increasing BrCl-flav concentrations resulting in an increased inhibitory activity. Moreover, the tested antifungal impaired plasma membrane organization, a cell structure which promotes also the virulence of the human fungal pathogen *C. albicans* [25] highlighting BrCl-flav therapeutic potential.

Biofilm formation also contributes to *Candida* antifungal resistance—a major problem faced by modern medicine. One solution could be the use of synergistic combinations of new molecules with traditional antifungals used in therapy to which *Candida* strains have already become resistant. Flavonoids in combination with fluconazole have been shown to display remarkable synergistic antifungal effects and are considered as reliable compounds for antifungal drug research and development [7]. In our study which involved a fluconazole resistant *C. albicans* clinical isolate, the MIC of fluconazole was 128-fold reduced in combination with BrCl-flav (concentrations ranging from 1.95 to 7.81 µg/mL), suggesting an important synergistic antifungal activity. We have to emphasize also that a combination of BrCl-flav and fluconazole in lower concentrations compared with individual MIC values showed important fungicidal effect with total kill after 48 h of incubation.

## 4. Materials and Methods

### 4.1. Chemicals and Fungal Strains

**Figure 9:**
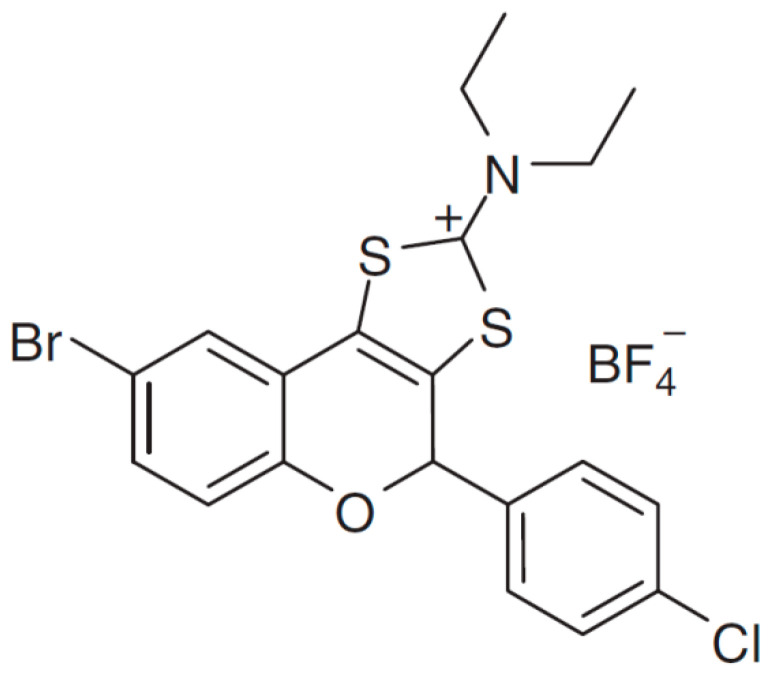
Structure of flavonoid BrCl-flav.

Tricyclic flavonoid BrCl-flav (Figure 9) was obtained as previously described [26]. The structure and purity (>99%) of the final compound have been established by NMR, MS, IR and elemental analysis. UV-Vis spectroscopy was employed to monitor the stability of BrCl-flav towards Sabouraud dextrose broth (SDB, Carl Roth, Karlsruhe, Germany), RPMI 1640 (Carl Roth) and phosphate buffer saline (PBS). The tricyclic flavonoid proved to be stable over a time span equivalent to the performed tests.

*Candida albicans*, *C. parapsilosis* and *C. krusei* were kindly provided by Dr Simona Matiut from the Praxis Clinical Laboratory (Iasi, Romania). *C. glabrata* strain was kindly provided by Dr M.N.L. Ngo-Mback (Laboratory for Phytobiochemistry and Medicinal Plants Studies, University of Yaounde I, Yaounde, Cameroon). The fungal strains were included in the microbial culture collection of the Faculty of Biology, University Alexandru Ioan Cuza of Iasi, with the following accession numbers: prxhif1-2018 (*C. albicans*), prx3-2018 (*C. parapsilosis*), prxbiof2-2018 (*C. krusei*) and cambio5-2017 (*C. glabrata*). *Candida krusei* (ATCC 6258) was used as reference strain for control. All clinical isolates were stored in 15% glycerol stocks at −80 °C. Prior to experiments, the organisms were transferred on Sabouraud dextrose agar (SDA, Carl Roth) and incubated 24 h at 37 °C. Subsequently, 15 mL of SDB were inoculated with one representative colony taken from SDA, cultured for 24 h (37 °C, 130 rpm) and used as source of inoculum for each experiment.

### 4.2. Assessment of Antifungal Activity

#### 4.2.1. Determination of Minimum Inhibitory Concentration (MIC) and Minimum Fungicidal Concentration (MFC)

The MIC values were determined by the broth microdilution method, in accordance with [6]. A concentration range of BrCl-flav between 0.12–250 μg/mL was tested with dimethyl sulfoxide (DMSO, Merck, Darmstadt, Germany) as solvent. DMSO was used as control with concentrations ranging from 25 to 0.012% (*v*/*v*) and nystatin as reference antifungal. *Candida krusei* ATCC 6258 was used as control strain. The inoculum was added into each well of a microplate (approximately 2.5 × 10^3^ CFU/mL final cell density), except the wells containing only SDB medium considered as blank. Inoculum and SDB medium were used as growth control. The lowest concentration showing no visible growth was considered as the MIC. A volume of 15 μL taken from each well with no visible growth was inoculated further on SDA to evaluate MFC. The MFC was considered the lowest concentration at which yeasts failed to grow in SDB supplemented with BrCl-flav and inhibited growth of the yeast after plating onto SDA [27].

#### 4.2.2. Fungal Growth Analysis

Effect of BrCl-flav on *Candida* spp. growth was assessed as we previously described [28], with some modifications. A volume of 100 μL from an overnight preculture was used to inoculate 20 ml SDB (final cell density approximately 2.5 × 10^3^ CFU/mL). In order to test fungistatic activity over time and to check the minimum inhibitory concentration values, the medium was supplemented with BrCl-flav to obtain final concentrations equivalent with ½ MIC, MIC, 2 × MIC. Inoculated SDB medium supplemented with DMSO without BrCl-flav at appropriate concentrations was used as control. All flasks were incubated on an orbital shaker (130 rpm) at 37 °C for 48 h. Samples were taken and growth rates were determined by measuring the optical density at 530 nm (OD530), using a DU 730 spectrophotometer (Beckman Coulter, Brea, CA, USA).

#### 4.2.3. Time-Kill Kinetic Assay

The killing rate of BrCl-flav was determined by measuring the reduction in the number of colony-forming units (CFU) per mL using the method of counting viable cells [29]. A volume of 100 μL from an overnight culture was added to 10 mL PBS (final cell density approximately 2.5 × 10^3^ CFU/mL) with various concentrations of BrCl-flav (½ MIC, MIC, 2 × MIC). Controls were prepared similarly using DMSO at appropriate concentrations. All flasks were incubated for 48 h at 37 °C. Samples were removed every 4 h up to 12 h and at 24, 48 h, serially diluted, inoculated onto SDA culture medium and incubated at 37 °C. Colonies were counted after 24 h and the viable cell number reported as CFU per mL was transformed into log10 values. The results were represented graphically obtaining a microbial death curve as a function of time. Fungicidal activity of BrCl-flav was considered when a reduction in microbial growth of ≥3 log10 in CFU/mL was recorded compared with the initial inoculum (99.9% killing of cells). Fungistatic activity was considered as a reduction in growth <3 log10 in CFU/mL from the initial inoculum (lower than 99.9%).

### 4.3. Sorbitol Binding Affinity Assay

The interference of BrCl-flav with the cell wall was assessed using the microdilution method presented above for determination of MIC. The MIC was recorded in the absence and presence of 0.8 M sorbitol after 48 h and it was considered as the lowest concentration at which no visible growth was observed [6].

### 4.4. Cell Membrane Permeability Test

The procedure described by Ma et al. [24] was used to analyze the fungal cell membrane permeability, with some modifications. *C. albicans* cells from an overnight culture were washed and suspended in PBS. The cell suspension (final cell density approximately 1 × 10^6^ CFU/mL) was incubated at 37 °C on an orbital shaker (130 rpm) for 48 h in presence of BrCl-flav at a concentration equivalent to MFC value (31.25 µg/mL). In order to observe a possible dose dependent effect of BrCl-flav against membrane structure, a higher concentration was also used (71.8 µg/mL—equivalent to 5 × MIC). Cells in PBS supplemented with DMSO served as control. Samples were taken at 1, 4, 6, 8, 24 and 48 h and stained with propidium iodide (PI, Carl Roth) for 15 min in the dark. The fluorescent cells were counted using a DM1000 LED fluorescence microscope (Leica, Wetzlar, Germany) and a I3 blue excitation range filter cube (BP 450 ± 490 nm band pass filter). At least five random, independent images were captured per sample and the ratio between fluorescent cells and total cells was calculated as percentage.

### 4.5. Hyphal Growth Test

Yeast to hyphal transition was performed following the procedure described by Wang et al. [30]. *C. albicans* cells from an overnight culture were incubated in RPMI 1640 at 37 °C in the presence of different BrCl-flav concentrations (MIC and 2 × MIC). Inoculated RPMI 1640 without flavonoid and supplemented with DMSO was used as control. Samples were taken at 6, 8, 10, 12, 24, and 48 h after inoculation and a simple staining using methylene blue (Sigma-Aldrich, Darmstadtcity, Germany) to distinguish between live and dead cells has been performed [31]. Yeast to hyphal transition was evaluated by microscopic observation using a Leica DM1000 LED microscope and scanning electron microscopy (Quanta 450, FEI, Thermo Fisher Scientific, Waltham, MA, USA). At least five random, independent images were captured per sample and used to count the number of individual yeast cells versus the number of hyphae. The hyphae inhibition rate (%) was calculated using the following Equation (1):

Hyphae inhibition rate = (hyphae % control − hyphae % BrCl-flav)/hyphae % control × 100(1)

### 4.6. Scanning Electron Microscopy (SEM)

*C. albicans* cell morphology after BrC-flav treatment was evaluated using SEM method, as we previously described [11]. An overnight culture was washed, centrifuged, suspended in PBS and incubated for 6 h in the presence of BrCl-flav at different concentrations (MIC, 2 × MIC and 5 × MIC) and DMSO as control. The samples were examined by SEM (Vega II SBH, Tescan, Brno, Czech Republic) at an acceleration voltage of 27.88 kV. Yeast to hyphal transition was investigated using a SEM system working in environmental mode (Quanta 450, FEI, Thermo Fisher Scientific, Waltham, MA, USA). The device is designed to be used with biological samples without the need to cover them with a conductive layer. The analyses were performed in low vacuum mode (at 100 Pa) in pure water vapor atmosphere with an electron acceleration voltage of 10 kV.

### 4.7. In Vitro Anti-Biofilm Activity Assay

The effect of BrCl-flav on *C. albicans*, *C. krusei* and *C. glabrata* biofilm-forming ability was determined as previously described [6]. Briefly, 250 µL of a cell suspension (1 × 10^3^ CFU/mL) in SDB was added into each well of a microtiter plate. The medium was supplemented with 6% of glucose and incubated at 37 °C for 48 h in presence of various concentrations of BrCl-flav. The wells containing only inoculated SDB supplemented with DMSO served as control. After an initial 6 h incubation, the culture medium was carefully removed and washed with distilled water to remove non-attached cells. A fresh medium was added and incubated to allow biofilm formation. After incubation, the wells were washed with distilled water to remove planktonic cells and crystal violet (Carl Roth) staining was performed [32]. A Beckman Coulter spectrophotometer was used to determine the optical densities (ODs) at a wavelength of 590 nm. Biofilm formation in the presence of BrCl-flav was expressed as a percentage of the control biofilm incubated in the absence of BrCl-flav (considered as 100%).

### 4.8. Checkerboard Assay

The effect of BrCl-flav in combination with fluconazole against *Candida* spp. resistant strains was assessed using the checkerboard microdilution method [6]. Two 96-well plates were used to obtain serial two-fold dilutions of the tested antifungal agents: the first microplate was used to dilute BrCl-flav in horizontal orientation and the second one was used to make dilutions of fluconazole in vertical orientation. The used concentrations of the tested antifungals were selected based on MIC values previously determined. All dilutions were made in SDB medium (50 µL per well). Fluconazole dilutions (50 µL) were transferred to the first plate to obtain different combinations of concentration with BrCl-flav. Subsequently, 100 µL of fungal suspension was added to each well (final cell density approximatively 10^3^ CFU/mL). SDB medium and inoculum served as control. The yeast growth was assessed visually after microplates incubation at 37 °C for 48 h. The lowest concentration showing no visible growth was considered as the MIC. To evaluate the synergistic effect, the fractional inhibitory concentration index (FICI) was calculated for each combination (Equation (2)):

FICI_AB_ = FIC_A_ + FIC_B_ (2)

The Fractional Inhibitory Concentration (FIC) of each agent (A or B) was calculated as a ratio of MIC when used in combination and MIC when used alone. FICI results for each combination (AB) were defined as synergy for FICI ≤ 0.5, additivity for 0.5 < FICI ≤ 1, indifference for 1 < FICI ≤ 4 and antagonism for FICI > 4. The effect of selected synergistic combinations was further assessed by time-killing curves.

### 4.9. Statistical Analysis

All experiments were performed in triplicate. The data are presented as mean (*n* = 3) ± S.E.M. The statistical evaluation of the results was carried out by Dunnett’s multiple comparisons test using the GraphPad Prism 9 software (GraphPad Software, Inc., La Jolla, CA, USA ). Differences between groups were considered significant when *p* < 0.05.

## 5. Conclusions

BrCl-flav is a synthetic flavonoid with an important fungicidal activity against *Candida albicans*, *C. parapsilosis*, *C. krusei* and *C. glabrata* clinical isolates at low concentrations. Our compound expressed synergic antifungal activity in combination with fluconazole and anti-virulence properties against *Candida* spp., inhibiting biofilm formation and yeast to hyphal transition. The mechanism of action is related to its ability to induce cell lysis by impairing cell membrane permeability and integrity. Taken together, these findings suggest that BrCl-flav has a considerable anti-*Candida* potential. Further studies are necessary to develop new antifungal drugs based on BrCl-flav.

## Figures and Tables

**Table 1:** Minimum inhibitory concentration and minimum fungicidal concentration of BrCl-flav against tested *Candida* species.

*Candida* sp. Strains	BrCl-Flav		DMSO	Fluconazole
	MIC (µg/mL)	MFC (µg/mL)	MIC (µg/mL)	MIC (µL/mL)
*C. albicans*	15.62	31.25	>250	1000
*C. parapsilosis*	15.62	31.25	>250	3.9
*C. krusei*	15.62	31.25	>250	62.5
*C. glabrata*	15.62	31.25	>250	125
*Candida krusei*	15.62	31.25	125	62.5
ATCC 6258				

MIC = minimum inhibitory concentration; MFC = minimum fungicidal concentration; the values are mean for at least three replicates.

**Table 2:** MIC values (μg/mL) of BrCl-flav in the absence and presence of sorbitol (0.8 M) against tested *Candida* strains.

*Candida* sp. Strains	MIC (μg/mL)	
	BrCl-Flav	BrCl-Flav + Sorbitol (0.8 M)
*C. albicans*	15.62	7.8
*C. parapsilosis*	15.62	7.8
*C. krusei*	15.62	15.62
*C. glabrata*	15.62	7.8

MIC = minimum inhibitory concentration; the values are mean for at least three replicates.

**Table 3:** Fractional inhibitory concentration indices (FICIs) of different BrCl-flav—fluconazole combinations against *Candida spp* strains.

*Candida* sp. Strains	MIC (µg/mL)				FICI	Interaction
	Alone		In Combination			
	BrCl-Flav	Fluconazole	BrCl-Flav	Fluconazole		
*C. albicans*	15.62	1000	15.62	0.16	1	IND
			7.81	7.81	0.50	SYN
			3.9	7.81	0.25	SYN
			1.95	7.81	0.12	SYN
*C. krusei*	15.62	62.5	15.62	0.12	1	IND
			7.81	31.25	1	IND
			3.9	31.25	0.75	ADD
			1.95	31.25	0.62	ADD
*C. parapsilosis*	15.62	3.9	15.62	0.12	1.03	IND
			7.81	1.95	1	IND
			3.9	1.95	0.75	ADD
			1.95	3.9	1.12	IND
*C. glabrata*	15.62	125	15.62	31.25	1.25	IND
			7.81	31.25	0.75	ADD
			3.9	62.5	0.75	ADD
			1.95	125	1.12	IND

MIC: Minimum inhibitory concentration; SYN: synergy (FICI ≤ 0.5); ADD: additivity (0.5 < FICI ≤ 1); IND: indifference (1 < FICI ≤ 4); the values are mean for at least three replicates.

## Data Availability

Data is contained in the article.
